# Physical activity and sedentary behavior can modulate the effect of the *PNPLA3* variant on childhood NAFLD: a case-control study in a Chinese population

**DOI:** 10.1186/s12881-016-0352-9

**Published:** 2016-12-01

**Authors:** Shuo Wang, Jieyun Song, Xiaorui Shang, Nitesh Chawla, Yide Yang, Xiangrui Meng, Haijun Wang, Jun Ma

**Affiliations:** 1 Institute of Child and Adolescent Health of Peking University, School of Public Health, Peking University Health Science Center, Beijing, 100191 China; 2 Interdisciplinary Center for Network Science and Applications (iCeNSA), University of Notre Dame, Notre Dame, IN 46556 USA; 3 Beijing Obstetrics and Gynecology Hospital, Capital Medical University,Beijing Maternal and Child Health Care Hospital, Beijing, 100026 China; 4 Centre for Population Health Sciences, Usher Institute of Population Health Sciences and Informatics, University of Edinburgh, Edinburgh, Scotland UK

**Keywords:** Non-Alcoholic Fatty Liver Disease, Physical activity, Sedentary behavior, Child and adolescent, Patatin like phospholipase containing domain 3 gene

## Abstract

**Background:**

The patatin like phospholipase containing domain 3 gene (*PNPLA3*) rs738409 C > G polymorphism, one of the most important gene polymorphisms involved in hepatic steatosis, has been reported to interact with different nutrients and dietary patterns on Non-Alcoholic Fatty Liver Disease (NAFLD), but no studies have focused on its interaction with physical activity or sedentary behavior. Therefore, this study aims at determining whether physical activity or sedentary behavior could modulate the effect of the *PNPLA3* variant on childhood NAFLD.

**Methods:**

A case-control study was conducted including 1027 Chinese children aged 7–18 years old (162 children with NAFLD and 865 children without). The anthropometric measurements, liver ultrasound examination, questionnaires and genotyping of the *PNPLA3* rs738409 polymorphism were performed.

**Results:**

Stratified analyses showed that the proportions of NAFLD increased with the G-allele number only in children who did not have enough physical activity (physical activity < 1 h/d) (OR 3.05, 95% CI 1.82–5.12, *P* < 0.001), and in children with a sedentary lifestyle (sedentary behavior ≥ 2 h/d) (OR 3.41, 95% CI 1.88–6.18, *P* < 0.001). Significant interactions on childhood NAFLD were found between the G-allele number in the *PNPLA3* rs738409 polymorphism and behaviors, including physical activity (*P* = 0.001), sedentary behavior (*P* = 0.010) and the combination of physical activity and sedentary behavior (*P* < 0.001).

**Conclusion:**

This is the first study to report the interaction between the *PNPLA3* rs738409 polymorphism and physical activity or sedentary behavior on NAFLD, providing new clues on the function of the *PNPLA3* gene, which will also be useful for future risk assessment and personalized treatment of NAFLD.

**Electronic supplementary material:**

The online version of this article (doi:10.1186/s12881-016-0352-9) contains supplementary material, which is available to authorized users.

## Background

The prevalence of childhood obesity has witnessed a substantial increase worldwide [1]. This has led to multiple co-morbid complications, including the Non-Alcoholic Fatty Liver Disease (NAFLD) that has become the most common form of liver diseases in children [2]. Worldwide, the prevalence of NAFLD among children ranges from 3% to 11% in the pediatric population [3] and between 9% and 37% in the general population [4].

It is suggested that the genetic background of the predisposition of NAFLD could be strong, and the patatin like phospholipase containing domain 3 gene (*PNPLA3*) is regarded as one of the most crucial genes in the development of hepatic steatosis [5]. The single nucleotide polymorphism (SNP) of *PNPLA3* rs738409 C > G (also known as *PNPLA3* I148M) is a missense variant, resulting in a cytosine to guanosine substitution which encodes for an isoleucine to methionine substitution at the amino acid position 148 (I148M) [5].

Previous studies have provided evidence of the main effect of the *PNPLA3* rs738409 polymorphism on NAFLD [6–8], and also its interaction with behavioral risk factors including total carbohydrate (specifically sugar) [9], high omega-6/omega-3 polyunsaturated fatty acids (PUFA) ratio [10], high sweetened beverage intake and low vegetable intake [11]. Recent studies demonstrated that lacking of physical activity and having too much sedentary behavior are also risk factors of NAFLD [12, 13]. However, in spite of those nutrients and dietary patterns, the interaction between the *PNPLA3* polymorphism and physical activity (PA), or sedentary behavior (SB) has not been studied.

In this study, we conducted analyses among 1027 Chinese children aged 7–18 years old (162 of them had NAFLD), in order to determine whether the *PNPLA3* rs738409 polymorphism interacts with physical activity and sedentary behavior on NAFLD.

## Methods

### Subjects

A case-control study was conducted and subjects were selected from 1093 individuals who participated in the study of Comprehensive Prevention project for Overweight and Obese Adolescents (CPOOA). As described before [14], the CPOOA subjects were recruited from children aged 7–18 years old in 5 elementary and middle schools of Haidian District of Beijing, comprising 637 overweight or obese children and 456 normal-weight children. All the obese individuals in the selected schools were recruited with their voluntary participation. The method of cluster sampling was adopted to recruit non-obese subjects from classes of each grade in the same schools. The ascertainment strategy for the study groups has been previously described in details [15, 16]. By asking medical history, we selected the subjects without any of the following conditions: alcohol consumption; a history of diseases or drugs (including herbal medicines) causing liver disease; common (HBV, HCV) or rare liver diseases, hepatic malignancies, infections biliary tract disease, any cardiovascular and metabolic diseases. Finally, 1027 participants having liver ultrasound examination and blood samples were included in the study. All participants provided their written informed consent, and for children under 16 year old, written informed consent was provided by their parents. The study was approved by the Ethic Committee of Peking University Health Science Center.

### Measurements

The 1027 children having data of liver ultrasound examination were classified into three categories—mild, moderate, and severe steatosis according to the following reference criteria [17]: 1) diffuse enhancement of near field echo in the hepatic region and gradual attenuation of the far field echo; 2) unclear display of intra-hepatic lacuna structure; 3) mild to moderate hepatomegaly with a round blunt border; 4) reduction of blood flow signal in the liver; and 5) unclear or non-intact display of envelop of right liver lobe and diaphragm. Patients meeting criterion 1 and any one of criteria 2–4 were classified as mild; patients meeting criterion 1 and any two of criteria 2–4 were classified as moderate; and patients meeting criteria 1, 5 and any two of criteria 2–4 were classified as severe. Data used in the staging system correlates well with histology, as demonstrated in a previous study [18]. All the examinations were performed by one experienced doctor, who was unaware of the patients’ clinical details and laboratory findings.

Physical activity and sedentary behavior were investigated using questionnaires. The questionnaires were completed by parents or guardians if the participant was ≤12 years old or by the subject if the participant was ≥13 years old. Sedentary behavior was determined by the time spent either on watching television or playing video/computer games per day during the last 7 days. It was then classified into categories of <2 h/d or ≥2 h/d according to the recommendation of a maximum of 2 h/d of television/video-watching and computer/video game-playing by the American Academy of Pediatrics [19]. Physical activity was determined by the time spent on physical activity per day during the last 7 days. It was then classified into categories of <1 h/d or ≥1 h/d because the WHO guidelines suggest children accumulating 60 min of moderate-to-vigorous physical activity daily [20].

### SNP genotyping

Genomic DNAs of subjects were extracted from blood leukocytes by the phenol-chloroform extraction method. Genotyping was conducted on MassARRAY System (Sequenom, San Diego, CA, USA). Primers, including a pair of amplification primers and an extension primer, were designed with Sequenom MassArray Assay Design Suite. A multiplex polymerase chain reaction was performed, and unincorporated double stranded nucleotide triphosphate bases were dephosphorylated with shrimp alkaline phosphatase followed by primer extension. The purified primer extension reaction was spotted on to a 384-element silicon chip (SpectroCHIP, Sequenom) and analyzed in the Matrix assisted laser desorption ionization time of flight mass Spectrometry (MALDI-TOF MS, Sequenom). The resulting spectra were processed with MassArray Typer (Sequenom) (http://www.sequenom.com). The genotyping call rate of the *PNPLA3* rs738409 polymorphism was 100%. All the experiments were done by investigators who were blind to the phenotypes.

### Statistical analyses

Statistical analyses were performed using the SPSS 18.0 software (SPSS Inc., Chicago, IL), and the Quanto software (University of Southern California, Los Angeles, CA). The status of NAFLD was transformed into a binary variable: children diagnosed as mild, moderate, and severe steatosis were NAFLD cases, and others were non-NAFLD controls. Differences in demographic and behavioral characteristics between NAFLD and non-NAFLD children were evaluated with t-tests (continuous variables) or Chi-square tests (category variables). The genotype data of the control group were tested for deviation from Hardy-Weinberg equilibrium. Multivariate logistic regression models with age, gender and BMI as covariates were used to calculate the odds ratios (OR) of the genetic variant or the interaction terms (genotype × behavior) for NAFLD. The polymorphism was analyzed under the additive model. A *P* value <0.017 after Bonferroni correction for multiple tests was considered statistically significant.

### Power calculation

As no studies have previously reported the interaction between the *PNPLA3* rs738409 polymorphism and physical activity or sedentary behaviors on NAFLD, we could only estimate the statistic power. Using the additive genetic model, at a two-sided significance level of *P* < 0.05, with the effect allele frequency of 0.40 and the prevalence of environmental factor of 0.40, the sample size in this study had over 75% power to detect an assumed effect of the gene-environment interaction (OR = 2.0).

## Results

### General characteristics of the study population

The demographic and behavioral characteristics of NAFLD cases and non-NAFLD controls are presented in Table 1. The subjects were around 11.5 years old with 55.9% of them being boys, and had an average BMI of 21.7 kg/m^2^. T-tests and Chi-square tests were conducted to evaluate the difference between NAFLD and non-NAFLD children for each of the characteristics. There was a marginal age difference between NAFLD children and controls (*P* = 0.060). Besides, NAFLD children had a larger proportion of boys (*P* < 0.001) and also a higher BMI (*P* < 0.001) as compared to non-NAFLD children. The percentage of SB ≥ 2 h/d was higher in NAFLD children (F = 10.181, *P* = 0.001), and logistic regression adjusted for age and gender indicated that sedentary time was positively associated with NAFLD (OR 1.64, 95% CI 1.14–2.36, *P* = 0.008). We did not find a significant association between physical activity time and NAFLD by the Chi-square test (F = 0.065, *P* = 0.798). After adjusting for age and gender in logistic regression models, the association between NAFLD and physical activity time was still not significant (*P* = 0.900). To examine whether the effect of physical activity or sedentary behavior on NAFLD was independent of each other, a multivariate logistic regression model was also constructed with independent variables including age, gender, physical activity and sedentary behavior. The result revealed that sedentary time was associated with NAFLD independently of physical activity (OR 1.64, 95% CI 1.14–2.36, *P* = 0.008), while there was no significant association between physical activity time and NAFLD (*P* = 0.895).

**Table 1:** Demographic and behavioral characteristics of NAFLD and non-NAFLD children

	Total	NAFLD	Controls	*P*	*P’*	OR
	(*n* = 1027)	(*n* = 162)	(*n* = 865)			
Age (years)	11.5 ± 2.9	11.8 ± 2.2	11.4 ± 3.0	0.060	·	·
Male (%)	574 (55.9)	115 (71.0)	459 (53.1)	<0.001	·	·
Body-mass index (kg/m^2^)	21.7 ± 4.3	26.8 ± 3.8	20.7 ± 3.6	<0.001	·	·
Physical Activity (PA)						
PA ≥ 1 h/d	531 (51.7)	76 (46.9)	415 (48.0)	0.798	0.900	1.02 (0.71–1.48)
PA < 1 h/d	459 (44.7)	70 (43.2)	365 (42.2)			
Sedentary Behavior (SB)						
SB < 2 h/d	628 (61.1)	75 (46.3)	509 (58.8)	0.001	0.008	1.64 (1.14–2.36)
SB ≥ 2 h/d	362 (35.2)	71 (43.8)	271 (31.3)			

Data are presented by mean (SD) or number(percentage). *P* values were calculated by t-tests or Chi-square tests

OR and *P’* values were for PA or SB calculated in logistic regression models adjusted by age and gender. The models were constructed for PA or SB separately

*NAFLD* Non-Alcoholic Fatty Liver Disease, *PA* Physical Activity, *SB* Sedentary Behavior

### Single polymorphism analysis

The genotype distribution of polymorphism in the control group was in Hardy-Weinberg equilibrium (*P* > 0.05). The results of single polymorphism analysis for association between NAFLD and the *PNPLA3* rs738409 polymorphism has been described in our previous study [8]. The distribution of the *PNPLA3* rs738409 genotype was shown in Table 2. There was a significant association between the G-allele number at the *PNPLA3* rs738409 and NAFLD when adjusted for age, gender and BMI (OR 1.57, 95% CI 1.15–2.16, *P* = 0.005), which means that possessing each G allele at rs738409 could increase the risk of childhood NAFLD by 57%.

**Table 2:** Interaction analyses of the *PNPLA3* rs738409 polymorphism and behavioral factors on childhood NAFLD

	Percentage of NAFLD children (number of NAFLD cases/number of the subgroup)						
	CC	GC	GG	ORa (95% CI)	*P*_a_	ORb (95% CI)	*P*_b_
Total	15.1 (60/398)	15.0 (74/492)	20.4 (28/137)	1.57 (1.15–2.16)	0.005		
Physical Activity (PA)							
PA ≥ 1 h/d	16.2 (31/191)	16.0 (38/237)	11.1 (7/63)	1.02 (0.63–1.66)	0.923	3.13 (1.57–6.24)	0.001
PA < 1 h/d	11.9 (19/160)	14.7 (31/211)	31.3 (20/64)	3.05 (1.82–5.12)	<0.001		
Sedentary Behavior (SB)							
SB < 2 h/d	14.2 (32/226)	9.8 (27/276)	19.5 (16/82)	1.22 (0.80–1.87)	0.363	2.47 (1.25–4.91)	0.010
SB ≥ 2 h/d	14.4 (18/125)	24.4 (42/172)	24.4 (11/45)	3.41 (1.88–6.18)	<0.001		
PA and SB							
PA ≥ 1 h/d & SB < 2 h/d	17.2 (22/128)	9.5 (14/147)	7.3 (3/41)	0.50 (0.26–0.96)	0.039	6.11 (2.79–13.37)	<0.001
PA < 1 h/d or SB ≥ 2 h/d	12.6 (28/223)	18.3 (55/301)	27.9 (24/86)	3.17 (2.02–4.96)	<0.001		

*NAFLD* Non-Alcoholic Fatty Liver Disease, *PNPLA3* The patatin like phospholipase containing domain 3 gene, *PA* Physical Activity, *SB* Sedentary Behavior

a*:* the *P* value of rs738409 in logistic model conducted in each behavioral level, including age, gender, BMI and rs738409 as independent variables

b*:* the *P* value of rs738409 × behavior in logistic model conducted for each behavior, including age, gender, BMI, rs738409, behavior, rs738409 × behavior as independent variables

The association effect of rs738409 on BMI was also tested, and no significant association was found in the enrolled subjects (β’ = -0.045, *P* = 0.113) or in any of the behavioral subgroups (*P* > 0.05). We found a marginal association of rs738409 with BMI in non-NAFLD children (β’ = -0.072, *P* = 0.0175), and the association was not significant in children with NAFLD (β’ = 0.083, *P* = 0.244). The results were shown in the Additional file 1: Table S1.

### Interaction analyses of rs738409 and behavioral factors on childhood NAFLD

**Fig. 1:**
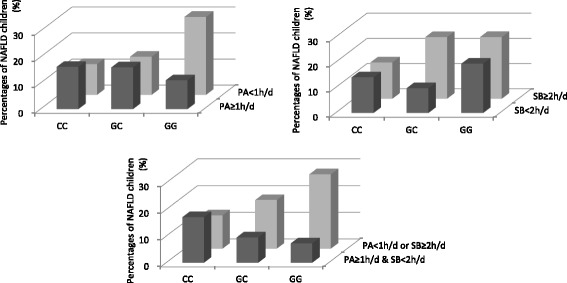
Percentages of NAFLD children in different genotypes of the *PNPLA3* rs738409 polymorphism and behavioral groups. NAFLD: Non-Alcoholic Fatty Liver Disease; *PNPLA3*: The patatin like phospholipase containing domain 3 gene; PA: Physical Activity; SB: Sedentary Behavior

Table 2 and Fig. 1 illustrate the percentage of NAFLD children in each subgroup divided by the *PNPLA3* rs738409 genotype and physical activity, sedentary behavior or the combination of physical activity and sedentary behavior). The percentage of NAFLD children increased with the G-allele number only among children who did not have enough physical activity (PA < 1 h/d) (OR 3.05, 95% CI 1.82–5.12, *P* < 0.001), and among children with a sedentary lifestyle (SB ≥ 2 h/d) (OR 3.41, 95% CI 1.88–6.18, *P* < 0.001). When we combined the physical activity and sedentary behavior, a similar trend revealed that among the inactive children (PA < 1 h/d or SB ≥ 2 h/d), the percentages of NAFLD increased significantly as children possess more G alleles (OR 3.17, 95% CI 2.02–4.96, *P* < 0.001), but among active children (PA ≥ 1 h/d & SB < 2 h/d) the association of rs738409 and NAFLD was not significant after Bonferroni correction (OR 0.50, 95% CI 0.26–0.96, *P* = 0.039).

Subsequently we tested the interaction term for each behavioral factor in multivariate logistic regression models including age, gender, BMI, rs738409, behavior and rs738409 × behavior as independent variables. For the behavioral factors, we first considered sedentary behavior and physical activity independently, and then we also tested the combination of two behaviors. The results revealed that the G-allele number of the *PNPLA3* rs738409 polymorphism interacted with physical activity on childhood NAFLD significantly (OR 3.13, 95% CI 1.57–6.24, *P =* 0.001), and also with sedentary behavior (OR 2.47, 95% CI 1.25–4.91, *P =* 0.010), and further with the combination of the two factors (OR 6.11, 95% CI 2.79–13.37, *P* < 0.001).

## Discussion

In this study, we found the interaction between the *PNPLA3* rs738409 polymorphism and physical activity or sedentary behavior on NAFLD among 1027 Chinese children aged 7–18 years old, by conducting the stratified analyses and the statistical tests for interaction terms. To the best of our knowledge, this is the first study reporting the interaction between the *PNPLA3* rs738409 polymorphism and physical activity or sedentary behavior on NAFLD.

The *PNPLA3* rs738409 polymorphism plays a crucial role in the development of NAFLD. It is reported that the *PNPLA3* 148 M allele encodes for an abnormal protein, which increases the risk of the accumulation of triglycerides in the liver [2, 21]. This gene is involved in the first hit of hepatic steatosis, and is related to the effect of other risk genes (such as *GPR120*) on the second hit resulting in the liver damage [2].

Previous studies have provided evidence of the main effect of the *PNPLA3* rs738409 polymorphism on NAFLD. A systematic review by meta-analysis on the influence of rs738409 on the NAFLD susceptibility proved that rs738409 had a strong effect on the fat accumulation, inflammation and fibrosis in the liver: when compared to CC homozygous, GG homozygous showed 73% higher lipid fat content, and also had 3.24-fold greater risk of higher necroinflammatory scores and 3.2-fold greater risk of developing fibrosis [6]. Studies in Chinese population also observed similar results. Li, et al. [7] reported that rs738409 was associated with the fatty liver degree in Chinese adults, with the risk allele frequencies of 0.32, 0.54, and 0.87 in mild, moderate, and severe cases, respectively. Shang, et al. [8] of our study group reported that the G-allele of the *PNPLA3* rs738409 polymorphism was associated with NAFLD (OR = 1.55, 95% CI: 1.13–2.11, *P* = 0.006).

The *PNPLA3* rs738409 polymorphism may play a crucial role in NAFLD developing via interaction with behavioral risk factors. Studies have indicated that the total carbohydrate (specifically sugar) [9], high omega-6/omega-3 polyunsaturated fatty acids (PUFA) ratio [10], high sweetened beverage intake and low vegetable intake [11] can influence the association between rs738409 and NAFLD. Davis, et al. [9] reported in a study of 153 Hispanic children of 8–18 years old that hepatic fat fraction (HFF) was influenced by a significant interaction between rs738409 and carbohydrate (genotype × carbohydrate, *P* = 0.04), specifically between genotype and total sugar intake (genotype × total sugar, *P* = 0.01). Santoro, et al. [10] conducted a study in 127 children and adolescents (aged 14.7 ± 3.3 years old, of different ethnic backgrounds) and found that HFF was influenced by the interaction of SNP and omega-6/omega-3 PUFA ratio (*P* = 0.002). Furthermore, interactions between the *PNPLA3* rs738409 polymorphism and the intake of sweetened beverage (*P* = 0.033) and vegetables (*P* = 0.038) on NAFLD were reported in a study consisting of 200 Italian obese children aged 10–13 years old by Nobili, et al [11]. Shen, et al. [22] conducted the interaction analysis in 920 Hong Kong Chinese (251 had NAFLD) but observed no significant interaction between rs738409 and the dietary pattern including energy intake, carbohydrate consumption, fat consumption or dietary fiber intake. Although evidence of different nutrients has been reported in terms of interaction analyses, we have not found studies focusing on the interaction between the *PNPLA3* polymorphism and physical activity or sedentary behaviors.

Recent studies have demonstrated that lacking of physical activity and having too much sedentary behavior are also risk factors of NAFLD [12, 13]. Hallsworth, et al. [12] observed that NAFLD patients spent nearly half an hour extra a day being sedentary (1318 ± 68 vs1289 ± 60 mins/day, *P* < 0.05) and walked 18% fewer steps (8483 ± 2926 vs 10,377 ± 3529 steps/day, *P* < 0.01) as compared to non-NAFLD controls, in a study consisting of 37 cases and 37 controls. Ryu, et al. [13] conducted a study among 139,056 Koreans (39,257 had NAFLD) and demonstrated that both long time of sedentary behavior and decreased time of physical activity could be associated independently with the increase of the NAFLD prevalence. The prevalence ratios(95% CIs) for NAFLD comparing sitting time of 5–9 h/d and ≥10 h/d to sitting time of <5 h/d were 1.04(1.02–1.07) and 1.09(1.06–1.11), respectively (*P* < 0.001); whereas the physical activity levels were negatively associated with the prevalence of NAFLD, with the prevalence ratios (95% CIs) for NAFLD which compared the ‘minimally active’ group and ‘health-enhancing physically active’ group to the ‘inactive’ group being 0.94(0.92–0.95) and 0.80(0.78–0.82), respectively (*P* < 0.001).

In this study, a positive association between sedentary behavior and NAFLD was revealed, and furthermore sedentary behavior could modify the effect of the *PNPLA3* rs738409 polymorphism on NAFLD. We did not find a significant primary association between physical activity and NAFLD, but this should not be considered to affect the importance of the interaction results, supported by several other related gene-environment interaction studies which also lack significant effects of behavioral factors. Davis, et al. [9] demonstrated the interaction between the *PNPLA3* rs738409 polymorphism and carbohydrate intake on HFF in which the carbohydrate intake was related to HFF only in the GG group, but not among the overall study population (when not split by genotype). Nobili, et al [11] reported the interactions between rs738409 and the intake of sweetened beverage (*P* = 0.033) and vegetables (*P* = 0.038), in which the consumption of specific food or dietary patterns were not directly associated with NAFLD when not considering the *PNPLA3* rs738409 genotype. Our results and the results of relative studies suggested that the rs738409 G × E interaction on NAFLD could be a ‘pure interaction’ instead of ‘quantitative’ of ‘qualitative’ interaction, which means the effect of one exposure is present only in the presence of the other, as explains by Hutter, et al. [23].

It has been emphasized that the *PNPLA3* rs738409 polymorphism interacted with obesity on the development of NAFLD. Shang, et al. [8] indicated that there was stronger association of rs738409 G-allele with NAFLD in obese children (OR = 1.85, 95% CI: 1.22–2.81, *P* = 0.004) than that in non-obese children (OR = 1.17, 95% CI: 0.71–1.92, *P* = 0.541), which suggested a ‘combined effect’ of rs738409 and obesity on the development of NAFLD. Another study conducted by Diehl, et al. [24] demonstrated that rs738409 interacts with the visceral adipose tissue volume in related to the liver fat content. Therefore, in this study we added the variable BMI in the logistic regression models as one of the covariates, when analyzing both the main effect of polymorphism on NAFLD and the effects of gene × behavior interaction, which could help to identify the effects independently of BMI. Besides, as the behaviors of physical activity and sedentary behavior are commonly thought to be risk factors of obesity, adjusting for BMI as the covariates in the statistical tests of interaction terms and also in the stratified analyses could ensure that the polymorphism interacts with the behavior itself, instead of with the BMI status related to the behaviors.

The association between rs738409 and BMI in non-NAFLD children (β’ = -0.072, *P* = 0.0175) should be noted. Although it was not significant as compared to the Bonferroni *P*-value threshold, the marginally negative association effect still suggested that in the non-NAFLD subgroup, children with more rs738409 non-risk alleles (C) could possibly have higher BMI. This should not be interpreted as a direct effect of rs738409 on BMI per se, but instead it could result from limiting the subjects to only non-NAFLD children. Since people with high BMI was supposed to have high risk of NAFLD [2], those subjects with high BMI but without NAFLD could possibly tend to possess more non-risk alleles that protect them against NAFLD, which could explain the marginally negative association effect in this subgroup.

In terms of the genetic model of the polymorphism, we only used the additive model. A study in 2010 conducted the evaluation of the risk of the *PNPLA3* rs738409 polymorphism associated with NAFLD, and suggested that the additive genetic model could best explain the effect of rs738409 on the susceptibility to develop NAFLD [6].

Several limitations of this study should also be noted. First of all, though we have found statistically significant interactions between the polymorphism and behaviors, the case-control study design means that we cannot assess the causal relation directly from the results. Secondly, the ideal approach for the diagnosis of NAFLD should be the histologic examination, which is the golden standard of the NAFLD diagnosis. Instead, we used the abdominal ultrasound examination to avoid doing invasive examinations on the children population. However, the diagnostic criteria using the ultrasound measurement have been previously proven to be capable of differentiating mild, moderate and severe steatosis [18], and ultrasound examinations have been used in a variety of studies [7, 8, 11–13]. Thirdly, the accurate food intake was not investigated in our study, such as the carbohydrate intake and the fatty acid intake which were reported by previous articles to interact with the *PNPLA3* rs738409 polymorphism on NAFLD [9, 10]. Another limitation of this study is that we did not determine the relation among the enrolled subjects, especially in terms of peer interaction. As reported by Salvy SJ, et al., peer social functioning (PSF) could influence children’s screen time and physical activity [25]. Further studies working on genetic-behavior interactions also need to consider the peer interaction factor. Finally, the limited sample size in this study should be noted, because gene-environmental interaction studies usually need a very large sample size to ensure reliable results in each subgroup. The ideal method to ensure the reliability of the results is the validation in a second cohort. Additional studies in a larger sample size with precise measurement of behaviors are needed to validate the relation among *PNPLA3*, physical activity, sedentary behaviors and NAFLD.

## Conclusion

In conclusion, we found the interaction between the *PNPLA3* rs738409 polymorphism and physical activity or sedentary behavior on NAFLD among 1027 Chinese children aged 7–18 years old. The risk of NAFLD children increased with the G-allele number only among children without enough physical activity (PA < 1 h/d) and among children with a sedentary lifestyle (SB ≥ 2 h/d). Further combination of physical activity and sedentary behavior showed that the risk of NAFLD increased with the risk allele number among the inactive children (PA < 1 h/d or SB ≥ 2 h/d), but decreased among active children (PA ≥ 1 h/d & SB < 2 h/d).

This is the first study reporting the interaction between the *PNPLA3* variant and physical activity or sedentary behavior on NAFLD, revealing that physical activity and sedentary behavior can modulate the effect of the *PNPLA3* variant on childhood NAFLD. It provided new clues on the function of the *PNPLA3* gene, and would also be useful for future risk assessment and personalized treatment of NAFLD.

## Additional files

Additional file 1: Table S1. Association effects of *PNPLA3* rs738409 on BMI in behavioral or NAFLD +/- subgroups. (DOCX 17 kb)
Additional file 2: Dataset of NAFLD status, rs738409 genotype and physical/sedentary behavior. Description of the dataset: In this dataset, each row represents a case and each column represents a variable. This dataset includes 7 variables: (a) NAFLD status (‘1’ = ‘NAFLD’, ‘0’ = ‘non-NAFLD’); (b) rs738409 genotype (‘0’ = ‘CC’, ‘1’ = ‘GC’, ‘2’ = ‘GG’); (c) gender (‘1’ = ‘male’, ‘2’ = ‘female’); (d) age (years); (e) BMI (kg/m2); (f) physical activity (‘0’ = ‘physical activity ≥ 1 h/day’, ‘1’ = ‘physical activity < 1 h/day’); (g) sedentary behavior (‘0’ = ‘sedentary behavior < 2 h/day’, ‘1’ = ‘sedentary behavior ≥ 2 h/day’). (SAV 42 kb)

## Abbreviations

NAFLD: Non-Alcoholic Fatty Liver Disease
OR: Odds ratios
PA: Physical activity
*PNPLA3*: Patatin like phospholipase containing domain 3 gene
PUFA: Polyunsaturated fatty acids
SB: Sedentary behavior
SNP: Single nucleotide polymorphism
