# Supplementary material for: Physical activity and sedentary behavior can modulate the effect of the PNPLA3 variant on childhood NAFLD: a case-control study in a Chinese population
Source: BMC Med Genet. 2016 Dec 1;17:90. doi: 10.1186/s12881-016-0352-9 (PMC5134284; doi:10.1186/s12881-016-0352-9)
Supplement: Additional file 1: Table S1. — Association effects of PNPLA3 rs738409 on BMI in behavioral or NAFLD +/- subgroups. (DOCX 17 kb) [file 12881_2016_352_MOESM1_ESM.docx]

Additional file 1: Table S1

| Table S1. Association effects of PNPLA3 rs738409 on BMI in behavioral or NAFLD +/- subgroups | | | |
| --- | --- | --- | --- |
|  | β' | β (95% CI) | *P* |
| Physical Activity (PA) |  |  |  |
| PA≥1h/d | -0.03 | -0.19 (-0.67-0.30) | 0.456 |
| PA<1h/d | -0.03 | -0.18 (-0.74-0.39) | 0.539 |
| Sedentary Behavior (SB) |  |  |  |
| SB<2h/d | 0.01 | 0.04 (-0.40-0.48) | 0.859 |
| SB≥2h/d | -0.09 | -0.60 (-1.27-0.06) | 0.075 |
| PA and SB |  |  |  |
| PA≥1h/d & SB<2h/d | -0.02 | -0.09 (-0.62-0.44) | 0.732 |
| PA<1h/d or SB≥2h/d | -0.03 | -0.22 (-0.69-0.25) | 0.357 |
| Disease Status |  |  |  |
| Non-NAFLD | -0.07 | -0.39 (-0.71-(-0.07)) | 0.0175 |
| NAFLD | -0.08 | -0.45 (-1.20-0.31) | 0.244 |

*PNPLA3*: The patatin like phospholipase containing domain 3 gene; NAFLD: Non-Alcoholic Fatty Liver Disease.

Age and gender were adjusted for in all models in Table S1.
